# Risk prediction of ICU readmission in a mixed surgical and medical population

**DOI:** 10.1186/s40560-015-0096-1

**Published:** 2015-06-26

**Authors:** Frida Kareliusson, Lina De Geer, Anna Oscarsson Tibblin

**Affiliations:** Department of Anaesthesiology and Intensive Care, Linköping University, Linköping, Sweden; Department of Medical and Health Sciences, Linköping University, Linköping, Sweden

**Keywords:** Intensive care units, Stability and workload index for transfer score, Simplified acute physiology score 3

## Abstract

**Background:**

Readmission to intensive care units (ICU) is accompanied with longer ICU stay as well as higher ICU, in-hospital and 30-day mortality. Different scoring systems have been used in order to predict and reduce readmission rates.

**Methods:**

The purpose of this study was to evaluate the Stability and Workload Index for Transfer (SWIFT) score as a predictor of readmission. Further, we wanted to study steps and measures taken at the ward prior to readmission.

**Results:**

This was a retrospective study conducted at the mixed surgical and medical ICU at Linköping University Hospital. One thousand sixty-seven patients >18 years were admitted to the ICU during 2 years and were included in the study. During the study period, 27 patients were readmitted to the ICU. Readmitted patients had a higher SWIFT score than the non-readmitted (16.1 ± 6.8 vs. 13.0 ± 7.5, *p* = 0.03) at discharge. The total ICU length of stay was longer (7.5 ± 7.5 vs. 2.9 ± 5.1, *p* = 0.004), and the 30-day mortality was higher (26 vs. 7 %, *p* < 0.001) for readmitted patients. Fifty-six percent of readmitted patients were assessed by the critical care outreach service (CCOS) at the ward prior to ICU readmission. A SWIFT score of 15 or more was associated with a significantly higher readmission rate (*p =* 0.03) as well as 30-day mortality (*p* < 0.001) compared to a score of ≤14.

**Conclusions:**

A SWIFT score of 15 or more is associated with higher readmission rate and 30-day mortality. The SWIFT score could therefore be used for risk prediction for readmission and mortality at ICU discharge.

## Background

The frequency of readmission within 72 h of intensive care unit (ICU) discharge is one of the most used and well-established measurements of quality in intensive care. Earlier studies have shown that readmitted patients have two to three times longer length of stay in the ICU than non-readmitted patients. In addition, readmitted patients have two to ten times higher risk of death than patients who are not readmitted [1–9]. Internationally, the readmission rate is 6–7 % [1, 2, 10]. Reasons for readmission are most commonly respiratory, cardiovascular and infectious complications. Studies have shown that these complications are related to the original disease in 19–65 % of the cases and due to new complications in 30–40 % of the cases [1, 2]. The readmission rate is higher at university hospitals than at non-university hospitals, and a risk factor that has been recognized is inadequate follow-up in the ward after ICU discharge. This could be due to a “critical care gap”, i.e. a difference in monitoring and care level between the ward and the ICU, resulting in suboptimal care for patients post-ICU. There are conflicting results whether the rate of unpredicted ICU readmission is higher for patients discharged at night [8, 11–13]. However, a correlation between a high occupancy in the ICU at the time of discharge and the risk of readmission has been shown [13]. In addition, readmitted patients have been shown to have a higher severity of illness, both at admission and discharge from the ICU than non-readmitted patients, with a higher Simplified Acute Physiology Score 3 (SAPS3) as well as Sequential Organ Failure Assessment (SOFA) score in readmitted patients than in those not readmitted [3, 6, 8, 14].

Previous studies have shown that patients admitted to the ICU have shown signs of treatable organ failure in the ward prior to ICU admission [15, 16]. In order to detect signs of deteriorating organ functions at an early stage, scoring systems have been developed for monitoring patients in the ward. One scoring system in clinical use is the Modified Early Warning Score (MEWS), where patients are scored based on their respiratory, circulatory and neurological state, renal function and body temperature [17–19]. Earlier data indicate that MEWS at ICU admission could predict ICU length of stay as well as ICU and 30-day mortality. However, MEWS score at ICU discharge was low and could not predict ICU readmission [20].

The Stability and Workload Index for Transfer (SWIFT) score is a scoring system specifically developed in order to predict readmission to the ICU. SWIFT consists of five different parameters: way of ICU admission, length of ICU stay, respiratory (two parameters) status and neurological status. SWIFT has been found to be able to predict unexpected readmission in both medical and surgical ICUs but has been studied less in a mixed ICU setting.

The aim of this study was therefore to evaluate the prognostic value of the SWIFT score in predicting ICU readmission and mortality in a mixed medical-surgical ICU population. An additional aim was to investigate the effectiveness of a modified SWIFT (M-SWIFT) score including renal function on predicting risk of ICU readmission. Our hypothesis was that the SWIFT score and M-SWIFT could be valuable tools in predicting readmission to the ICU.

## Methods

Ethical approval for this study was provided by the Regional Ethics Committee in the South East of Sweden, Linköping, Sweden on the 12th of December 2012 (number 2012/412-31). Informed consent from patients was waived on the basis that this was not an intervention study.

A retrospective cohort study was conducted at the mixed surgical-medical ICU at the University Hospital, Linköping, Sweden. The hospital is a tertiary teaching hospital with a total of 600 beds and 41,000 admissions yearly. The ICU is an eight-bed ward with 600 admissions yearly. The ICU manages critically ill surgical and medical patients, except for patients undergoing cardiothoracic surgery. The ICU also provides a 24 h, 7 days a week, critical care outreach service (CCOS). The CCOS consists of an anaesthetist or intensive care physician and an intensive care specialist nurse. The criteria for calling the CCOS are MEWS score >4, and MEWS has been used as a monitoring tool in the hospital since 2008. All patients >18 years admitted to the ICU between 1 January 2011 and 31 December 2012 were included in this study.

All data regarding the critical care admission for all patients are registered in digital medical records, in paper ICU charts, later scanned as attachments to the digital records, and in the Swedish Intensive Care Registry (SIR). Information about age, gender, time of admittance and discharge, length of ICU stay, source of admission, admitting ward, accepting ward after discharge, SAPS3 on admittance, SOFA score at discharge, comorbidities, diagnoses and treatment strategy as well as ICU occupancy was collected from SIR.

The SWIFT score consists of five variables: original source of admission, length of ICU stay, last measured partial pressure of oxygen in arterial blood (PaO_2_)/fraction of inspired oxygen (FiO_2_) (mmHg) ratio, last measured PaCO_2_ (mmHg) and Glasgow Coma Scale (GCS) at discharge, all seen in Table 1. The SWIFT score was calculated for all patients discharged to a ward. The PaO_2_/FiO_2_ ratio and PaCO_2_ were extracted from the last arterial blood gas taken before ICU discharge. The GCS at discharge was collected from the paper ICU chart. In cases where GCS was missing in the charts, it was calculated from discharge entries in the medical records. The MEWS score at discharge was also calculated based on information in ICU charts and medical records. For some patients, the MEWS score at discharge was already reported in the ICU chart, and in these cases, this value was used. A M-SWIFT score was calculated for all patients discharged to a ward. M-SWIFT was calculated using the SWIFT score, with an additional assessment of renal function. Patients with a urine output <500 mL/day and/or a creatinine level >170 μmol/L (equalling a SOFA renal score >2) received five additional points. Readmission was defined as an unexpected readmission to the ICU within 72 h after ICU discharge. For these patients, medical records were thoroughly studied, regarding the time after discharge until readmission. The alteration in MEWS score at the ward, if the CCOS had been called upon, interventions at the ward, treatment strategies and reason for readmission were documented.

**Table 1:** The stability for workload and transfer score

Variable	SWIFT point
Original source of this ICU admission	
Emergency department	0
Transfer from a ward or outside hospital (any type of nursing unit)	8
Total ICU length of stay (duration in days)	
<2	0
2–10	1
>10	14
Last measured PaO_2_/FiO_2_ ratio (during this ICU admission)	
>400	0
<400 and >150	5
<150 and >100	10
<100	13
Glasgow Coma Scale at time of ICU discharge	
>14	0
11–14	6
8-10	14
<8	24
Last arterial blood gas PaCO2	
<45 mmHg	0
>45 mmHg	5

### Statistical analysis

The comparison of data such as age, length of ICU stay, SAPS3, SOFA score, SWIFT and M-SWIFT scores as well as numerical variables included in the SWIFT and SOFA scores was made with unpaired Student’s *t* test. Pearson’s chi-squared test was used to compare categorical data such as gender, original source of admission, ward at admission, mortality, treatment strategy and the presence of comorbidity. The MEWS score was compared with a two-sample Mann-Whitney *U* test. A *p* value <0.05 was considered significant. Data analysis was performed in STATA version 12.1 (StataCorp. LP, College Station, Texas, USA) and SPSS version 20 (IBM corp, Armonk, New York, USA).

## Results

### Study population

During the study period, 1244 patients were admitted to the ICU. One hundred seventy-four patients (14 %) were aged <18 years. Three patients were defined as postoperative patients and were therefore excluded from the study. Of the total adult population, 140 patients (13 %) were discharged to another ICU. Ninety-seven patients (9 %) died during the ICU stay. Thirty-four patients (3 %) were discharged to another hospital, and 10 patients (1 %) were discharged to their home. A total of 786 patients (74 %) were discharged to a ward in the hospital and 53 of these (7 %) had treatment strategies, i.e. at discharge, treating physicians had decided not to readmit the patient to the ICU in case of deterioration. The number of patients at risk of readmission was therefore 733.

### ICU admission

Out of 1067 adult patients admitted to the ICU, 332 patients (31 %) were admitted from a ward, 281 patients (26 %) from the emergency department, 244 patients (23 %) from the operating theatre, 101 patients (9 %) from another ICU, 98 patients (9 %) from a ward at another hospital and 11 patients (1 %) were obstetric patients (Table 2). Five hundred sixty-three patients (53 %) were surgical, 458 patients (43 %) were medical, and 46 patients (4 %) were oncological patients. The mean length of stay at the ICU were 3.7 days (95 % CI 3.3–4.0) (Table 2).

**Table 2:** Demographic characteristics of studied patients

Variables	*n* = 1067	95 % CI
Age, year, mean (SD)	57 (19)	56–58
Gender, *n* (%)		
Male	613 (57)	
Female	454 (43)	
Original source of admission, *n* (%)		
Emergency department	281 (26)	
Other ICU	101 (9)	
Other hospital	98 (9)	
Operating theatre	244 (23)	
Ward	332 (31)	
Delivery ward	11 (1)	
Ward at admission, *n* (%)		
Medical wards	458 (43)	
Surgical wards	563 (53)	
Oncologic wards	46 (4)	
Severity of illness		
SOFA score, mean (SD)	4.0	3.8–4.2
SAPS3, mean (SD)	55.1	54.1–56.1
SWIFT score, mean (SD)	14.0	13.4–14.6
MEWS, mean (SD)	1.9	1.8–2.0
Treatment strategy, *n* (%)	114 (11)	
Outcomes		
ICU mortality, *n* (%)	97 (9)	
30-day mortality, *n* (%)	226 (21)	
Length of ICU stay, day, mean (SD)	3.7	3.3–4.0
ICU Readmission, *n* (%)	27 (2.5)	

*CI* confidence interval

### ICU readmission

**Fig. 1:**
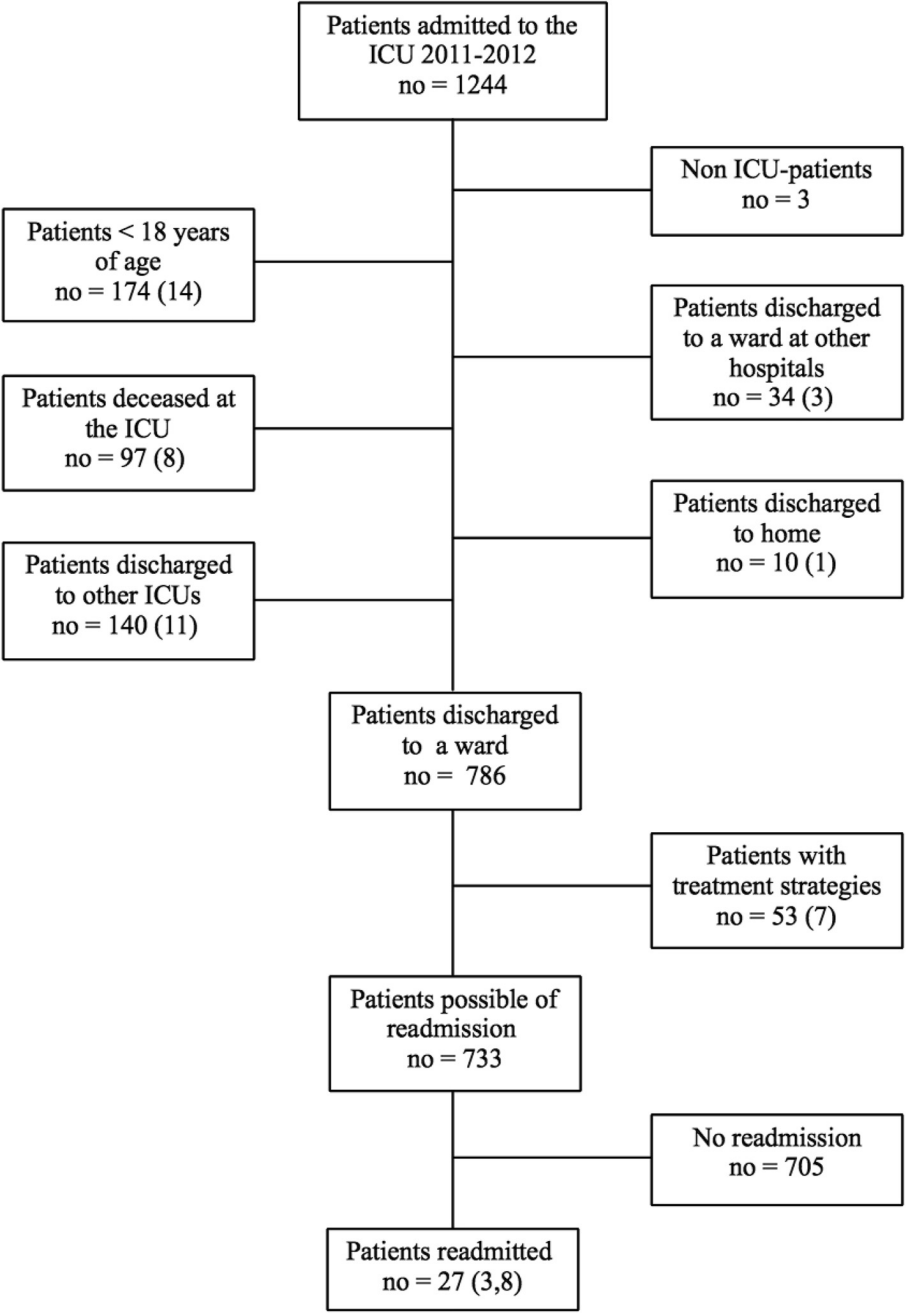
Patient discharge flowchart

Twenty-seven patients (2.5 %) were readmitted to the ICU within 72 h after discharge (Fig. 1). When excluding all patients discharged to another ICU, to another hospital, to their home and those with treatment strategies, the readmission rate was 3.8 %. Readmitted patients were significantly older than patients who were not readmitted (65 ± 12 vs. 54 ± 19, *p =* 0.005) (Table 3). In addition, readmitted patients had a higher SOFA score (4.4 ± 2.8 vs. 3.0 ± 2.2, *p* = 0.01) and a higher MEWS at discharge (2.3 ± 1.3 vs. 1.8 ± 1.2, *p =* 0.03) than non-readmitted patients (Table 4). Readmitted patients tended to more often be women, more often been admitted from a hospital ward and more often to be surgical patients than non-readmitted, however, not statistically significant (Table 3). Readmitted patients were more often immunocompromised, 1 patient (4 %) compared to 13 patients (2 %) (*p* = 0.05). Kidney failure was also more common among readmitted patients (6 (23 %) vs. 54 (8 %), *p* =0.05) (Table 3). At discharge, readmitted patients had a significantly higher SWIFT score (16.1 ± 6.8 vs. 13.0 ± 7.5, *p* = 0.03) than non-readmitted. When comparing different parameters of the SWIFT score, readmitted patients had a significantly lower PaO_2_/FiO_2_ ratio (*p =* 0.03) than non-readmitted patients. PCO_2_ did not differ between the readmitted and non-readmitted patients when comparing means. However, readmitted patients were significantly more likely to have a PCO_2_ > 45 mmHg than non-readmitted patients (*p =* 0.04). Readmitted patients also had a significantly higher M-SWIFT score than non-readmitted patients (17.6 ± 7.3 vs. 13.5 ± 7.7, *p* = 0.007) (Table 4). The median (interquartile range (IQR)) ICU occupancy during the study period was 78 % (66–90 %). When patients who were later readmitted were discharged, the median (IQR) ICU occupancy was 88 % (75–100 %, *p* = 0.002).

**Table 3:** Characteristics of readmitted and non-readmitted patients

Variables	Readmission (*n* = 27)		No. of readmission (*n* = 705)		*p* value
		95 % CI		95 % CI	
Age, years, mean	64.7	60–70	54.2	53–56	0.005
Gender, *n* (%)					0.38
Male	400 (57)		13 (48)		
Female	305 (43)		14 (52)		
Original source of admission, *n* (%)					0.42
Emergency department	4 (15)		210 (29)		
Other ICU	1 (4)		36 (5)		
Other hospital	2 (7)		42 (6)		
Operating theatre	6 (22)		192 (25)		
Ward	14 (52)		216 (31)		
Delivery ward	0 (0)		9 (1)		
Ward at admission, *n* (%)					0.26
Medical wards	7 (26)		288 (41)		
Surgical wards	19 (70)		404 (57)		
Oncologic wards	1 (4)		13 (2)		
Comorbidity, *n* (%)					
Immunodeficiency	1 (4)		13 (2)		0.05
Chronic obstructive pulmonary disease	1 (4)		46 (6)		0.59
Kidney failure	6 (23)		54 (8)		0.005
Chronic	2 (7)		10 (1)		
Liver failure	0 (0)		6 (1)		0.64
Heart failure	0 (0)		22 (3)		0.36
Malignancy	4 (15)		84 (12)		0.59
Sepsis	3 (12)		75 (11)		0.88
Outcome					
Total length of ICU stay, days, mean (SD)	7.5 (7.5)		2.9 (5.1)		0.004
Length of ICU stay, primary admission, mean (SD)	3.0 (5.2)		2.8 (4.1)		0.88
ICU mortality, *n* (%)	6 (22)		na		
30-day mortality, *n* (%)	7 (26)		50 (7)		<0.001
Treatment strategy, *n* (%)	6 (22)		na		

*CI* confidence interval

**Table 4:** Severity of illness scores in readmitted and non-readmitted patients

Variables	Readmission (*n* = 27)		No. of readmission (*n* = 705)		*p* value
		95 % CI		95 %CI	
SAPS3, mean (SD)	56.0 (11.6)	51.4–60.6	50.5 (14.4)	49.5–51.6	0.50
MEWS, mean (SD)	2.3 (1.3)		1.8 (1.2)		0.03
SWIFT score, mean (SD)	16.1 (6.8)	13.4–18.9	13.0 (7.5)	12.4–13.6	0.03
Length of ICU stay, dy, mean (SD)	3.0 (5.2)	1.2–4.5	2.8 (4.1)	2.6–3.4	0.88
Source of admission *n* (%)					0.09
Emergency	4 (15)		210 (30)		
Other source	23 (85)		495 (70)		
PaO_2_/FiO_2_ mmHg, mean (SD)	287 (109)	244–330	338 (119)	329–348	0.03
GCS at discharge	14.7 (0.5)	14.5–14.9	14.7 (1.2)	14.6–14.8	0.91
PCO_2_ mmHg, mean (SD)	41.0 (8.0)	37.8–44.2	39.7 (8.2)	39.1–40.3	0.42
PCO_2_ mmHg, *n* (%)					0.04
<45	18 (67)		526 (83)		
>45	9 (33)		111(17)		
Modified SWIFT score, mean (SD)	13.5 (7.7)		17.6 (7.4)		0.007
SOFA score, mean (SD)	4.4 (2.8)	3.3–5.6	3.0 (2.2)	2.8–3.2	0.001
Respiration	2.1 (1.1)		1.4 (1.1)		0.002
Coagulation	0.8 (1.1)		0.4 (0.8)		0.07
Liver	0.3 (0.6)		0.3 (0.7)		0.86
Cardiovascular	0.3 (0.7)		0.3 (0.6)		0.78
CNS	0.2 (0.5)		0.2 (0.6)		0.57
Renal	0.8 (1.1)		0.4 (0.8)		0.06

*MEWS* Modified Early Warning Score, *SAPS3* Simplified Acute Physiology Score 3, *SOFA* Sequential Organ Failure Assessment score, *SWIFT* Stability and Workload Index for Transfer score

### Readmitted patient’s data on ward

**Fig. 2:**
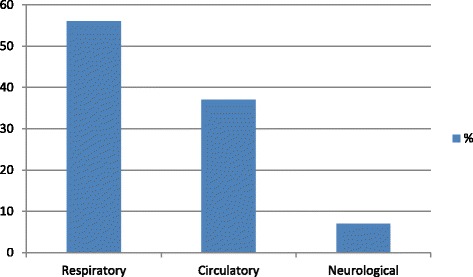
Reasons for ICU readmission

At discharge, three (11 %) of the 27 readmitted patients had a MEWS score of at least four, which is the criterion for calling CCOS at our hospital. For these three patients, CCOS were called two to three times after ICU discharge. Fifteen readmitted patients (56 %) had a MEWS score >4 at the ward and therefore reached the criteria for a CCOS consultation. Nine (60 %) of these 15 patients were assessed by the CCOS. Of the 12 patients that had MEWS <4, 6 patients (50 %) had a CCOS consultation. Eight of the readmitted patients (30 %) showed confusion and/or anxiety at the ward. At the ward, 16 patients (59 %) had respiratory interventions such as suction of the airways, CPAP or inhalations. Thirteen patients (48 %) had circulatory interventions such as extra fluids and/or diuretics. Two patients (7 %) had infectious interventions with a change in antibiotic treatment. Ten of the 27 patients (37 %) were readmitted due to circulatory complications, 15 patients (56 %) to respiratory complications and 2 patients (7 %) to neurological complications (Fig. 2). Of the 10 patients readmitted for circulatory reasons, 5 (50 %) had a circulatory intervention at the ward and 3 (30 %) had a respiratory intervention. Of the 15 patients readmitted due to respiratory reasons, 12 (80 %) had respiratory interventions and 8 (53 %) had circulatory interventions at the ward.

### Outcome

The total length of ICU stay was significantly higher among readmitted patients (7.5 ± 7.5 vs. 2.9 ± 5.1, *p* =0.004) (Table 3). ICU mortality was significantly higher in readmitted patients compared to the whole ICU population (21 % vs. 9 %, *p =* 0.02). The mortality after 30 days was also significantly higher in readmitted patients 7 (26 %) compared to 50 (7 %) of the non-readmitted, *p* < 0.001 (Table 3). The 30-day mortality was 21 %. Patients discharged to a ward without treatment strategies with a SWIFT ≥15 had a significantly higher readmission frequency as well as 30-day mortality rate compared to patients with a SWIFT ≤14 (Table 5). A M-SWIFT ≥15 compared to a M-SWIFT ≤14 was also associated with a significant higher frequency of readmission and 30-day mortality (Table 5).

**Table 5:** Outcome in relation to SWIFT score

Variables	SWIFT ≤14	SWIFT = ≥15	*p* value
	(*n* = 466)	(*n* = 195)	
Readmission, *n* (%)	14 (3)	13 (7)	0.03
30-days mortality, *n* (%)	26 (6)	33 (17)	<0.001

*SWIFT* Stability and Workload Index for Transfer score

## Discussion

In this study, we found that patients readmitted to the ICU had a significantly higher SWIFT score as well as M-SWIFT at ICU discharge than non-readmitted patients. We found that readmitted patients had higher MEWS and SOFA scores than non-readmitted patients, indicating a higher severity of illness at discharge. Furthermore, a SWIFT as well as M-SWIFT score ≥15 was associated with higher rate of readmission and 30-day mortality. This indicates the need for caution when discharging patients above this cut-off.

In line with previous results, we found a significant difference in respiratory parameters between readmitted and non-readmitted patients at discharge, with decreased PaO_2_/FiO_2_ ratio in the readmitted [11, 21, 22]. In addition, when studying the individual parameters in SOFA, the respiratory score was significantly higher in readmitted patients. Also, 56 % of readmissions were due to respiratory reasons. Thus, respiratory functions seem to be central to the risk of ICU readmission, and our results also suggest that patients are discharged without sufficiently stable respiratory functions. In addition, our results indicate a possible weakness in wards regarding specific respiratory support after critical care.

In addition to a reduced PaO_2_/FiO_2_ ratio, we found that readmitted patients had a significantly higher rate of kidney failure, acute as well as chronic, than non-readmitted patients. Three previous studies found similar results regarding chronic renal disease [2, 6, 23]. One earlier study found that low urine output and abnormal creatinine levels were a risk factor for readmission [22]. Since kidney injury is a well-described and strong prognostic indicator for poor outcome after intensive care, a scoring system for risk of ICU readmission should logically include a parameter for renal function. Based on these data, we decided to add a renal parameter to the SWIFT score. We found that readmitted patients had a higher modified SWIFT score than non-readmitted patients, indicating that renal function is an important factor for readmission. We believe that a modified SWIFT, including renal function, would increase the accuracy of the score in predicting readmission. Further and larger studies are needed to test this hypothesis. Also, a prospective study assessing the rate of readmissions before and after the introduction of SWIFT score or modified SWIFT score would indeed be interesting.

Readmitted patients had a higher severity of illness when discharged from the ICU, indicating that these patients may have been discharged somewhat prematurely. This correlates with earlier studies showing that 22–42 % of unexpected ICU readmissions in patients with a reduced physiological reserve are due to premature discharge [22, 24]. The reason behind early discharge is beyond the scope of this study, but the lack of ICU beds has, in other studies, been presented as an important cause [6, 9, 11, 23, 25]. There are no formal ICU discharge criteria at the ICU studied, but our results indicate, in line with these previous studies, that ICU occupancy may have contributed to the decision to discharge the patients.

In order to reduce unexpected admissions to the ICU by optimizing the treatment of patients in the ward, CCOS has been introduced in many hospitals worldwide. The CCOS, often consisting of an intensivist and an intensive care nurse, can be called upon by ward staff when a patient meets specific physiological criteria. The introduction of these teams has been shown to reduce unexpected ICU admissions [26, 27], and even to reduce ICU readmissions [28, 29] and mortality in readmitted patients [30]. Another study showed that patients discharged from the ICU and later readmitted were more likely to need a CCOS consultation, and patients meeting the CCOS criteria had 25 times higher risk for readmission [31]. The CCOS criterion at Linköping University Hospital is primarily a MEWS score >4. Interestingly, only 60 % of the patients meeting this criterion were assessed by the CCOS, corresponding to results found in another study [31]. The reason for this is unclear, but possible reasons may be that CCOS consultations are not formally obliged and may have been replaced by a direct physician to physician contact, or even overlooked. Also, some patients may have been readmitted after an emergency incident at the ward, not preceded by a CCOS consultation. Furthermore, 50 % of patients having MEWS <4 had a CCOS consultation, which might indicate that the CCOS was called upon for general worries of the patient or concerns regarding parameters not considered in the MEWS score. The ability of the ward to consult the CCOS team for other reasons than formal CCOS criteria may have contributed to the low readmission rate seen in this study.

A number of studies have evaluated different scoring systems for predicting readmission and death in ICU patients. In this study, we have focused on the SWIFT score, the MEWS score and the SOFA score as well as the M-SWIFT score, all of which were significantly higher in patients who were readmitted than in those who were not. The different scores use different parameters, but all overlap to some degree. The discharge of a patient may therefore be reconsidered if the patient scores high in all scales and when having a SWIFT score of 15 or more in particular.

Internationally, the ICU readmission rate is 6–7 % [1, 2, 10]. In contrast, the readmission rate in this study was low (2.5 %). Differences in ICU admission and discharge criteria as well as treatment options in the wards are possible reasons behind these differences, and in practice, a 0 % readmission rate is scarcely a realistic goal. However, our results indicate that among readmitted patients are some in whom readmission could have been avoided if their severity of illness had been more thoroughly assessed before discharge or if monitoring and treatment at the ward had been optimised. In view of this, we believe that the ICU readmission rate could be decreased further, possibly by a more thorough assessment of the risk of readmission in all patients discharged, followed by a formalised CCOS consultation in those at risk of readmission.

### Limitations

This study has several limitations. First, this was a single-centre study, and its generalizability is therefore limited. Second, the population of readmitted patients was small compared to international data. Third, this was a retrospective observational study. Therefore, there was no control group when studying factors in the ward prior to readmission.

## Conclusions

In conclusion, we found that readmitted patients had a higher SWIFT as well as M-SWIFT score. Readmitted patients were more severely ill than non-readmitted patients at discharge and may have been discharged from the ICU too early. Readmitted patients had longer ICU length of stay and higher 30-day mortality, and SWIFT score could be used as a predictor of 30-day mortality. ICU discharge should therefore be reconsidered in patients with a SWIFT score of at least 15. Larger studies are needed to prospectively investigate the SWIFT and M-SWIFT scores as predictors of readmission and to further study the period at ward prior to readmission.

## Abbreviations

CCOS: critical care outreach service
CI: confidence interval
FiO_2_: fraction of inspired oxygen
GCS: Glasgow Coma Scale
ICU: intensive care unit
MEWS: Modified Early Warning Score
M-SWIFT: Modified Stability and Workload Index for Transfer score
PaCO2: partial pressure of carbon dioxide in arterial blood
PaO_2_: partial pressure of oxygen in arterial blood
SAPS3: Simplified Acute Physiology Score 3
SOFA: Sequential Organ Failure Assessment score
SWIFT: Stability and Workload Index for Transfer score
